# Improving Pre-Exposure Prophylaxis Provision as Part of Routine Gynecologic Care Among Black Cisgender Women (Project PrEP4Her): Protocol for the Implementation of an Intervention

**DOI:** 10.2196/58976

**Published:** 2025-03-14

**Authors:** Maira Sohail, Lynn Matthews, Audra Williams, Mirjam-Colette Kempf, Desiree Phillips, Hannah Goymer, Bernadette Johnson,, Michael Mugavero, Latesha Elopre

**Affiliations:** 1 Department of Medicine University of Alabama at Birmingham Birmingham, AL United States; 2 Department of Nursing University of Alabama at Birmingham Birmingham, AL United States; 3 School of Public Health University of Alabama at Birmingham Birmingham, AL United States

**Keywords:** Black, cis-Gender women, PrEP, pre-exposure prophylaxis, South, HIV, gynecology

## Abstract

**Background:**

Although HIV pre-exposure prophylaxis (PrEP) has been proven to be an effective prevention tool in decreasing HIV transmission, achieving adequate PrEP uptake has remained a challenge among Black cisgender women living in the Southern United States. Gynecology clinics, which provide primary health care services for many cisgender women, have the potential to be an ideal setting for the integration of PrEP services.

**Objective:**

We designed an intervention, PrEP4Her, which aims to implement PrEP service delivery at gynecology clinics in Alabama, the United States, as part of routine reproductive and sexual health care visits to improve PrEP engagement rates among Black cisgender women.

**Methods:**

Guided by the information gathered on (1) factors impacting PrEP implementation at gynecology clinics, including key barriers and facilitators to PrEP implementation and potential strategies to address the identified barriers (in-depth interviews with the gynecology care team), (2) structural barriers and provider-level barriers to PrEP implementation (cross-sectional study among gynecologists), and (3) implementation strategies on how to integrate PrEP services into routine gynecology care (in-depth interviews and focus groups with Black cisgender women), a multicomponent implementation strategy, tailored for Black cisgender women, was developed to integrate PrEP in routine women’s health visits (ie, PrEP4Her). To determine the efficacy of the program, we will measure implementation outcomes, reach (increase in the absolute number of Black cisgender women receiving PrEP prescriptions), effectiveness (increase in the proportion of PrEP prescriptions over time), and adoption (proportion of team members willing to implement PrEP4Her) using the RE-AIM (Reach, Effectiveness, Adoption, Implementation, and Maintenance) framework. In addition, acceptability (the extent to which providers and Black cisgender women feel PrEP4Her to be acceptable—in-depth interviews); Feasibility (appropriateness of PrEP4Her for a larger, full-scale trial—the Feasibility of Intervention Measure scale); and fidelity (the degree to which PrEP4Her program was implemented as designed—electronic survey with patients) will also be assessed.

**Results:**

The qualitative and quantitative data from the gynecology care team and the qualitative data from Black cisgender women were collected from August 9, 2022, to April 19, 2023, and were integrated through joint displays to identify major themes. The combined findings provided a comprehensive understanding of factors that were fundamental in the development and refinement of PrEP4Her implementation. The PrEP4Her was implemented from January 29, 2024, to August 16, 2024. The information gathered is being used to assess PrEP4Her efficacy (based on reach, effectiveness, adoption, acceptability, feasibility, and fidelity).

**Conclusions:**

Upon completion of our research, our interdisciplinary team, which includes experts in infectious diseases, implementation science, community-engaged research, and psychology, will be primed to lead a multisite type III implementation trial for PrEP service delivery at gynecology clinics across the Southern United States.

**International Registered Report Identifier (IRRID):**

DERR1-10.2196/58976

## Introduction

Black people only constitute 13% of the US population but account for more than half of the new HIV diagnoses [1]. These disparities are more pronounced in the South [2], where the highest acquisition and mortality rates are documented among Black people [3]. In response to these inequities, the US federal agencies are working in a coordinated manner to end the HIV Epidemic with a mandate to focus on populations facing HIV inequities and geographic hot spots, with prioritization to increase utilization of effective biomedical prevention tools like HIV pre-exposure prophylaxis (PrEP), that reduces HIV transmission with consistent use [4-7]. The US state of Alabama has been prioritized for End the HIV Epidemic efforts due to high HIV incidence and prevalence rates. In Alabama, where the rate of HIV diagnoses in 2020 was 14.1 per 100,000 people, most new diagnoses occurred among Black men and women [8]. In addition, in 2021, Alabama had a PrEP-to-Need ratio of 5.55, with the highest unmet need for PrEP occurring among the Black community (PrEP-to-need ratio among Black vs White individuals: 2.94:11.30) [9].

When focusing on cisgender women, who account for approximately one-fifth of new HIV diagnoses in the United States, only 8% of the 227,010 women with a PrEP indication are currently receiving PrEP prescriptions [10-13]. This low PrEP uptake is similar in Alabama, where only 7% of the PrEP clientele comprised of cisgender women in a university-affiliated infectious diseases-led PrEP Clinic [14]. Among cisgender women, Black cisgender women are even more disproportionally impacted by the HIV epidemic, currently accounting for 54% of all new HIV diagnoses among cisgender women, nationally [15]. These disparities are mirrored in Alabama’s HIV incidence, where Black cisgender women are 9 times more likely to be diagnosed with HIV compared with White cisgender women [16]. With Black cisgender women in the Southern United States being disproportionately impacted by the HIV epidemic, approaches to increase PrEP uptake among this population present an opportunity to ameliorate these inequities.

Previous studies among Black cisgender women have found PrEP uptake barriers to occur at multiple levels (ie, individual, interpersonal, and Meso levels) [17]. Some individual-level barriers include a lack of awareness of PrEP and concerns around PrEP-related stigma, including fear of being labeled as HIV-positive with PrEP use, limiting PrEP uptake in this population. In addition, some provider-level barriers have also been discovered, such as a lack of knowledge on current PrEP clinical care guidelines, misconceptions related to insurance coverage, and provider bias preventing culturally appropriate PrEP-related discussions [18]. In addition, providers’ stereotypic beliefs or prejudices (ie, having higher levels of color-blind racial attitudes) lead to a decreased willingness to counsel and prescribe Black cisgender women PrEP [19]. As such, overcoming current inequities requires innovative strategies to engage Black cisgender women in PrEP service delivery tailored to their preferences.

Of the total HIV diagnoses among Black cisgender women, the majority occur in those of reproductive age [20]. There is strong evidence to support that 70% of women in the United States are seeking routine annual care for reproductive health and nearly half access contraceptive services [21,22]. Safety-net programs created for cisgender women to receive reproductive health care, like family planning clinics, have been evaluated in Southern states as sites for PrEP implementation [23,24]. However, a major barrier to PrEP uptake is low provider knowledge on the delivery of PrEP services. Interventions focused on improving provider knowledge alone have fallen short on seeing huge gains in PrEP uptake among women [25-28]. In Addition, previous studies have shown that even with increased PrEP access in clinical care settings that specialize in women’s health care, PrEP uptake has been low and providers have struggled in optimizing these spaces for PrEP service delivery [22-27,29]. Gynecologists have been shown to be ideal PrEP service delivery candidates, as national data supports higher willingness to prescribe PrEP among gynecologists compared with general practitioners [30]. Furthermore, 81% of patients seen by gynecologists are of reproductive age (18-44 years old), which also reflects the age range among cisgender women more likely to acquire HIV [31]. In line with this, our previous work among Black cisgender women in the state of Alabama also revealed they preferred PrEP services to be delivered by gynecologists in a clinical care setting [32].

Informed by compelling preliminary data, we designed an implementation study to determine the requisite steps and strategies for the implementation of PrEP services into routine gynecological care visits at a university-affiliated clinic in Birmingham, Alabama, which provides services to women with low income across the state [33]. Our gynecological pilot site is a large center that sees over 17,000 patients (40% Black cisgender women) annually, with the majority being of reproductive age, who reside in both urban and rural areas of Alabama. This paper outlines the protocol for this multicomponent implementation strategy, which evaluated key individual-level, inner-setting, and process-level determinants for the implementation of PrEP service delivery in gynecology clinic settings. The intervention aimed to develop and refine a multicomponent implementation strategy (PrEP4Her) that integrated PrEP in a gynecology clinic, which was then piloted to deliver PrEP for Black cisgender women attending routine gynecologic care visits. The PrEP4Her intervention is aimed at improving PrEP engagement among Black cisgender women in order to reduce the HIV disparity among this population.

## Methods

### Study Setting

The PrEP4Her was implemented at the University of Alabama at Birmingham gynecological Continuity clinic.

### Development of the Protocol

To explore perceived barriers and facilitators for integrating PrEP into routine gynecological care, qualitative and quantitative data were collected. In-depth interviews were conducted with gynecologists, practice managers, medical assistants, nurses, and pharmacists at our gynecological clinical site via teleconference. The interviews covered topics, such as factors effecting PrEP implementation at gynecological clinics, key barriers or facilitators to PrEP implementation, potential strategies to address the identified barriers, and any emerging topic areas for subsequent exploration. In addition, existing PrEP coverage services and programs for uninsured Black cisgender women were discussed with medical service providers, case managers, and social workers. After each interview, interviewers documented field notes, including emerging topic areas for subsequent exploration. The qualitative data were analyzed by an expert qualitative researcher using the NVivo (Lumivero) qualitative data management software. First, a preliminary codebook was developed, which included inductive codes, that emerged from the data, and pattern codes, that connected concepts to one another. The lead qualitative analyst along with a team of 2 researchers applied these codes to a subset of interview transcripts and adjusted the code definitions until an adequate interrater reliability was achieved. The finalized codebook was then used to analyze the remaining transcripts, noting major themes. Finally, data matrices were created to visually represent associations between key concepts and patterns in the data.

In addition to interviews, a cross-sectional study was conducted in collaboration with the Alabama Section of the American College of Obstetricians and Gynecologists and the university’s Center for Women’s Reproductive Health in the Department of Obstetrics and Gynecology to understand barriers to future state-wide implementation that needed to be measured during this pilot study. Surveys were conducted with English-speaking, currently practicing gynecologists or advanced practitioners at gynecological clinics (n=39), to evaluate structural barriers to PrEP as well as any potential provider-level barriers. The surveys documented information on sociodemographic, HIV, and PrEP knowledge [34,35], HIV- and PrEP-related stigma [36,37], and questions ascertaining current routine testing and screening practices. The data from the surveys reported findings on willingness to prescribe PrEP by the independent variables (sociodemographics: race and ethnicity, clinic name, gender, years of practice, clinic resources, characteristics of populations served; knowledge: PrEP and HIV; stigma: PrEP and HIV; and practice: sexual history, sexual transmitted infections testing, and sexual transmitted infections treatment) as well as willingness to prescribe PrEP (primary outcome) and the capability of the clinical-setting for providing PrEP services (secondary outcome).

To explore the desired PrEP educational materials and communication strategies from gynecologic care teams, in-depth interviews, and focus groups were conducted with Black cisgender women. The inclusion criteria for the in-depth interviews and focus groups were the same and included (1) self-reported HIV-negative status, (2) being Black cisgender women, (3) ability to speak in English, and (4) age of 18 to <54 years (the choice of age range is based on the current HIV epidemiology reporting highest HIV incidence among cisgender women aged <54 years). An informed consent was signed by eligible Black cisgender women before the interview and focus group participation. The semistructured interviews covered topics such as sexual health, education needed to increase awareness and knowledge, stigma, and communication with the care team. The qualitative data were analyzed in a similar manner as described above by the expert qualitative data team.

To develop and refine a multicomponent implementation strategy (PrEP4Her) that aimed to integrate PrEP in gynecology clinics, an intervention mapping technique was used [38]. First, key barriers to PrEP implementation among Black cisgender women and providers were reviewed from the data collected with the PrEP4Her community advisory board (CABs, with the full description provided below). Next, using existing knowledge of components identified from studies that implemented PrEP at family planning clinics in the South, an initial list of components was created. Although family planning clinics may have differences from our gynecological setting in terms of the type of care providers and resources, they share a mission for providing reproductive and sexual health services, which may result in overlap with strategies needed for our research population. Using the knowledge gained through the reported findings, the team modified their list of components for PrEP4Her in a way that was more suited toward our gynecological clinics. Table 1 and Textbox 1 outline the preliminary name, definition, and operationalization of components based on current feedback from the PrEP4Her CAB, established by Proctor et al [39-41]. The team then mapped the implementation strategy and linked it to the desired implementation outcomes. This was followed by developing a content-specific implementation strategy. After implementation strategy materials were refined, appropriate operational steps needed to integrate PrEP into the clinical site were reviewed. JS, who has expertise in implementation science and has previously worked with family planning clinics in Southern settings to improve HIV prevention among Black cisgender women, also helped with PrEP4Her implementation.

**Table 1:** Specification of components of the implementation strategy (“PrEP4Her”).

Implementation strategy	Number 1	Number 2	Number 3	Number 4	Number 5	Number 6
Actor	Medical assistant or care provider	Study staff or PrEP^a^ expert	PrEP expert	PrEP expert and information technician	PrEP expert and graphic designer	PrEP expert
Action	Charts evidence-based sexual history questionnaire in the medical record	Provides 1.5-hour training on HIV, risk, and PrEP indications and care	Creates easily accessible pocket cards with indications for PrEP	Creates templates in electronic medical records for PrEP visits and order sets	Develops informational content to include in educational materials tailored to Black cisgender women	Develops 30-minute training tutorial on how to communicate with patients about sexual health and PrEP, tailored to Black cisgender women
Target	Patient	Providers or staff	Provider	Provider	Patient	Patient
Temporality	Medical assistant (before every patient encounter with a provider) or provider (beginning of every visit)	1 month before PrEP4Her being implemented at our pilot site	Continuously carried by providers and displayed in shared workspaces	1 month prior to PrEP4Her being implemented with instructions on how to access placed in shared workspaces and sent through email communications	Placed in waiting areas and private examination rooms, accessible during every visit and replenished by study staff members	1 month before PrEP4Her being implemented and presentation accessible in the shared workspace as well as emailed to all providers
Dose	5-minute screener at every visit	1.5-hour training occurring once before 6-month pilot	As needed by providers during clinical care	As needed by providers during charting	Available during the entire 6-month pilot for every clinic	Once before 6-month pilot and then available as needed

^a^PrEP: pre-exposure prophylaxis.

**Pre-exposure prophylaxis 4Her components and outcomes.**

**Figure 1:**
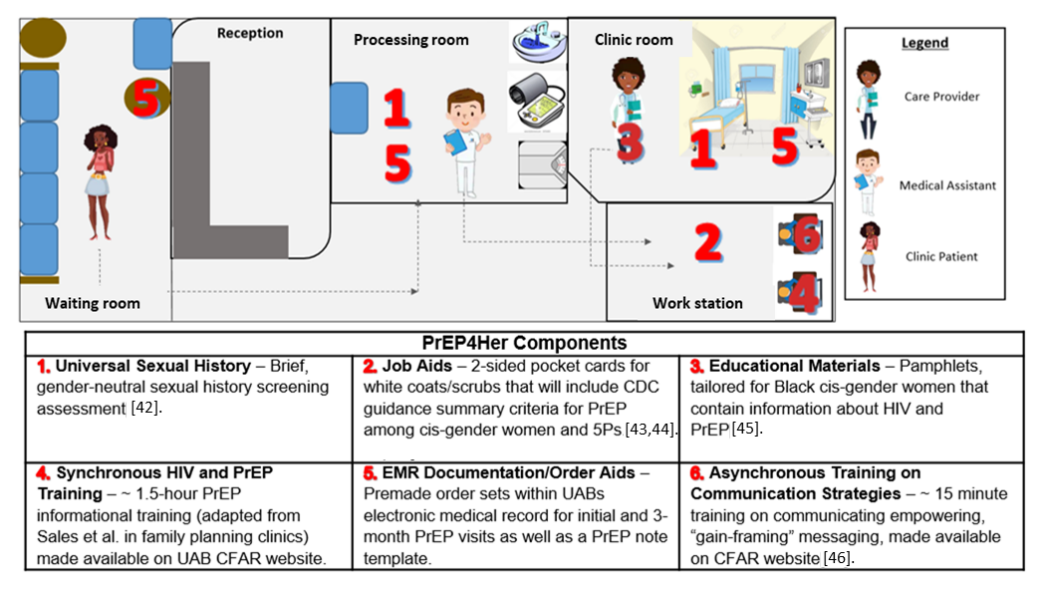
Pre-exposure prophylaxis implementation science research logic model. CAB: Community Advisory Board; GYN: gynecology; PrEP: pre-exposure prophylaxis.

- Named pre-exposure prophylaxis 4Her components
- Universal sexual health screening for gynecological patients
- Synchronous training of gynecological care team on HIV, pre-exposure prophylaxis indication, pre-exposure prophylaxis care (for both daily and long-acting injectable regimens), and stigma
- Pre-exposure prophylaxis indication job aids
- Electronic medical record documentation aids, including pre-templated notes and order sets
- Educational materials for Black cisgender women on HIV and pre-exposure prophylaxis
- Asynchronous training on effective communication strategies with patients about sexual health and pre-exposure prophylaxis
- Implementation outcomes affected
- Figure 1. for detailed description of how each implementation strategy maps to implementation outcomes. In short, we will be evaluating the following proximal implementation outcomes: reach, adoption, fidelity, acceptability, and preliminary effectiveness. Distal outcomes will not be evaluated in this study.
- Justification
- Implementation strategy components selected for this study have been selected based on observed, researched, or hypothesized barriers based on a review of the literature. These components may be subject to change based on findings in Aim 1 and feedback from our community advisory board.

### Conceptual Framework

This multiphase study will be evaluated using the RE-AIM (Reach, Effectiveness, Adoption, Implementation, and Maintenance) conceptual framework and will link our multicomponent implementation strategy to proximal implementation outcomes. Figure 1 depicts the research logic model for the implementation of the PrEP4Her study. The PrEP4Her components box shows the implementation strategies (A to F): A: carrying out the universal sexual health screening for gynecological patients in the medical record; B: providing training on HIV, PrEP indication, PrEP care, and stigma for the gynecological providers; C: creating job aids such as pockets cards to easily identify those with a PrEP indication; D: creating aids for electronic medical record documentation such as creating templates for PrEP-related visits and order sets; E: developing educational material tailored toward Black cisgender women; and F: developing training tutorials on communication methods with patients about sexual health and PrEP, tailored toward Black cisgender women. Moreover, the change of mechanisms box shows how the strategies in the PrEP4Her components box are anticipated to impact the implementation outcomes (the superscripts indicate the implementation strategies that are expected to cause the change). Finally, the proximal implementation outcomes box outlines the expected outcomes that will be assessed as per the RE-AIM framework; the superscripts again indicate the PrEP4Her components that will impact each of the outcomes. The project is expected to improve the conditions deemed as potential barriers to PrEP usage among Black cisgender women, ultimately achieving the desired outcomes of increased PrEP uptake among Black cisgender women. Our study describes the planning and piloting stages of the implementation research logic model, evaluated by the RE-AIM framework, in which we will work with key informants and Black cisgender women to assess the reach, adoption, and preliminary effectiveness of integrating PrEP services within a gynecological clinic in Birmingham, Alabama, with the ultimate goal of increasing uptake of PrEP.

### The Role of CABs

Two CABs, one for Black cisgender women (N=5) and one for gynecology practitioners (N=7), have worked and will continue to work with the study researchers. The CABs are led by a graduate-level researcher, who is also a part of this project. The CABs previously provided help with the interpretation of the qualitative findings and in selecting and refining components of the implementation strategy (PrEP4Her). Currently, the CABs meet monthly and advise on how to disseminate study results. At least 10 meetings were or will be held during the study, with a shared decision-making structure. Detailed notes will be documented to evaluate the meeting processes.

### Piloting PrEP4Her to Deliver PrEP for Black Cisgender Women Attending Routine Gynecologic Care Visits

**Figure 2:**
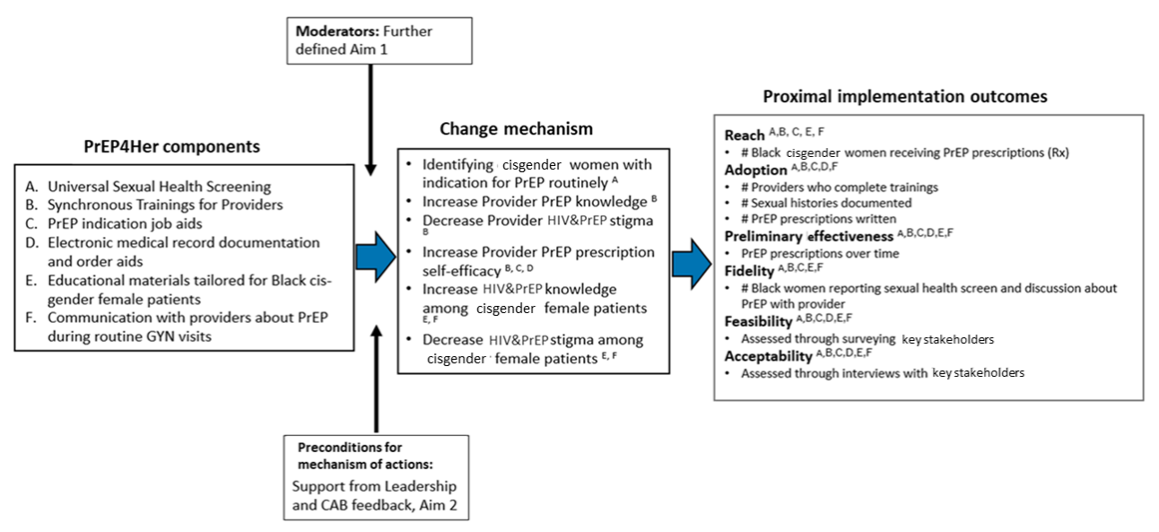
Pre-exposure prophylaxis 4Her components [42-46]. Image created by LE. CDC: Centers for Disease Control and Prevention; CFAR: Center for AIDS Research; PrEP: pre-exposure prophylaxis; UAB: University of Alabama at Birmingham.

PrEP4Her is a multicomponent implementation strategy, which was evaluated through a 1-arm pilot at a university-affiliated gynecological clinic. We chose a 1-arm trial, as PrEP was not routinely offered at gynecological clinics in Alabama and there were only 3 Black cisgender women on PrEP at the university-affiliated PrEP clinic at the study start, which meant a comparison between other gynecological clinics or between our PrEP clinics would have had limited meaning. Upon completion of PrEP4Her development, gynecological staff members, practice managers, and clinicians received training as well as implementing job and documentation aids within the clinic. In addition, educational materials were placed in all areas where patients are seen within the clinic. Figure 2 outlines the workflow for PrEP4Her along with the detailed description of each component. Implementation outcomes for PrEP4Her were monitored over a 6-month time frame (Jan-Aug 2024). Of the RE-AIM components, only reach, effectiveness, and adoption will be evaluated as a part of this study. In addition to the 3 components of RE-AIM, we will also evaluate the acceptability, feasibility, and fidelity of the trial.

### Reach Outcome

Reach in RE-AIM captures the ability to engage the target population in the intervention. In PrEP4Her, this will correspond to the absolute number of Black cisgender women who receive a PrEP prescription. In addition, the proportion of Black cisgender women prescribed PrEP among those who had a PrEP indication, will also be evaluated. PrEP indication will be based on the sexual risk screener (component 1 in Figure 2) documented in the chart during patient visits. For this study, PrEP indication will be defined based on the Centers for Disease Control’s guidance summary criteria. According to this guidance, cisgender women not living with HIV meet at least one of the following criteria are considered to have a PrEP indication: (1) HIV-positive sex partner not virally suppressed, (2) bacterial STI in the past 6 months (ie, gonorrhea or syphilis), (3) more than one sex partner, (4) inconsistent condom use, (5) sex for exchange of goods, or (6) being in a high-prevalence network [44]. PrEP prescription data will be obtained from the university's electronic medical records visit data during the pilot period. Although measuring the number of PrEP discussions with Black cisgender women by electronic medical record audit was also considered an important aspect to examine reach, it was deemed to add little value due to the anticipated inconsistencies in documentation by providers in reporting PrEP discussion in their clinic notes. However, we have incorporated this element in our surveys with Black cisgender women, evaluating their experiences with providers in receiving PrEP health counseling.

#### Effectiveness Outcome

Effectiveness in RE-AIM evaluates the effect of the intervention on the primary outcome as well as effect modification. Effectiveness of PrEP4Her will be determined by the increase in PrEP prescriptions over time. In addition, patient factors associated with PrEP prescription will be evaluated [47]. PrEP prescription data from the electronic medical records will be extracted at baseline, at 3 months, and at 6 months of PrEP4Her implementation, to evaluate effectiveness. Moreover, information on patient demographics (ie, age, race, ethnicity, and insurance status) will also be obtained. Using this data, frequencies for PrEP prescription will be reported overall and for each independent variable at baseline, 3 months, and 6 months.

#### Adoption Outcome

Adoption in the RE-AIM determines the ability of the setting’s staff members to adopt the intervention. This will be determined by measuring the proportion of key informants within the pilot site who were willing to initiate the PrEP4Her implementation strategy. This will be assessed by evaluating the training and sexual history documentation.

### Training

A website will be created as a part of this study, to aid in easy access of training materials for providers and staff members. During initial training, the number of providers and staff members who attend the meetings will be documented. In addition, viewing training on the website will be captured through collecting para data (tracking clicks).

#### Acceptability Outcome

Acceptability is defined as the extent to which key informants felt PrEP4Her was acceptable (ie, providers delivering the intervention and Black cisgender women receiving the intervention). This will be measured by conducting key-informant interviews. The sample will include 15-20 participants, with at least 10 providers, and approximately 10-15 Black cisgender women seen during the 6-month pilot period. Recruitment of providers will be carried out directly from the pilot study, whereas recruitment of Black cisgender women will be done through flyers and direct referrals from clinical staff members. Interview guide questions will be grounded in a theoretical framework of acceptability by Sekhon et al [48]. Interviews will be conducted virtually to allow for flexibility, using a secure system. Interviews will be digitally audio recorded, professionally transcribed, and deidentified. A preliminary codebook will be developed consisting of a priori deductive codes (based on the theoretical framework), inductive codes (emerging from the data), and pattern codes (connecting concepts to one another). The analysis of these data will be carried out in a similar manner as described above by our expert qualitative team.

#### Feasibility

Feasibility of the intervention will determine if PrEP4Her is appropriate for a larger, full-scale implementation trial. This information will be captured using the validated scale, Feasibility of Intervention Measure [49], through surveys conducted before interviews among key informants. The survey will include straightforward items such as “[PrEP4Her] was appealing to me,” “[PrEP4Her] seemed suitable,” and “[PrEP4Her] seemed easy to implement,” which respondents will rate on a 5-point Likert scale. Descriptive statistics will be calculated to determine feasibility.

#### Fidelity

Fidelity to the intervention protocol will be assessed as the degree to which the PrEP4Her implementation strategy was implemented as designed [50]. To measure fidelity, all patients seen during the 6-month pilot period will be given a flyer by the staff upon clinic checkout with information to take a brief 5-minute electronic survey. Recruitment will end once 300 surveys have been completed. While this intervention is tailored to Black cisgender women in terms of developed educational materials and provider communication, implementation strategy components are universal to all patients and, therefore, targeted sampling of only Black cisgender women will not be conducted. The brief electronic survey will include the following questions: (1) Did your provider (or other clinical staff members) take a history asking about your sexual activities and health? (2) Did you see educational materials during your visit about HIV prevention and a prevention method called PrEP (3) Did you speak with a provider about PrEP? (4) For those who spoke with a provider about PrEP, would you please describe the conversation and how you felt about communicating with your provider about this prevention strategy? As the survey contains both close-ended and open-ended questions, data will be analyzed both, quantitatively (descriptive statistics) and qualitatively (inductive and pattern coding).

The findings from the pilot will provide the necessary information to refine PrEP4Her for broader dissemination and future multisite implementation studies.

### Ethical Considerations

#### Human Subject Ethics Review Approvals

The following study was approved by the University of Alabama at Birmingham’s institutional review board (IRB#300008345) on December 2, 2022.

#### Informed Consent

An informed consent was collected and documented from all gynecological interview participants. The quantitative data used for this study was covered under the study’s institutional review board and was secondary in nature that data was abstracted from available deidentified electronic medical records.

#### Privacy and Confidentiality

All data were deidentified before being analyzed.

#### Compensation Details

All CAB members received a US $50 stipend per meeting. Each interviewee and focus group participant was compensated with US $50. Patients who will complete the electronic survey determining Fidelity will receive a compensation of US $20 after completion.

## Results

**Figure 3:**
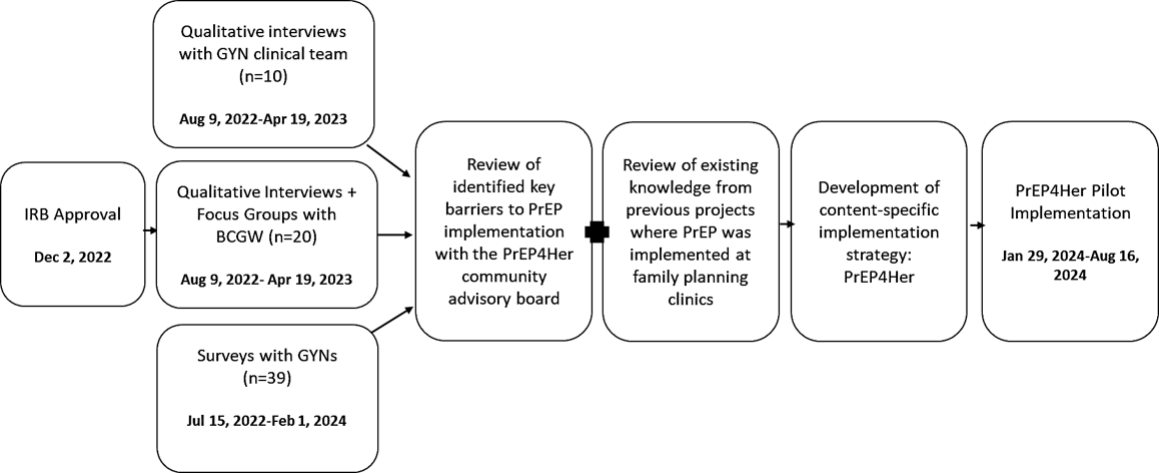
PrEP4Her study timeline BCGW: Black cisgender women; IRB: institutional review board; GYNs: gynecologists; PrEP: pre-exposure prophylaxis.

Figure 3 outlines the PrEP4Her study timeline. The in-depth interviews with the gynecological care team were conducted between August 9, 2022, to April 19, 2023. A total of 10 in-depth interviews were conducted (40% were attending or training physicians). In addition, the cross-sectional study used data collected from surveys with gynecologists from July 15, 2022, to February 1, 2024 (n=39; 80% female). Moreover, the in-depth interviews and focus groups with black cisgender women (n=20) were conducted between Aug 9, 2022, to Apr 19, 2023. The qualitative findings from in-depth interviews with the gynecological care team (physicians, nurses, and medical assistants) and in-depth interviews and focus groups with women patients (64% black) highlighted a gap in care delivery for women (manuscript in the process of publication), suggesting additional training of providers may be beneficial. This was added as one of the strategies to improve PrEP uptake under the PrEP4Her implementation.

The qualitative and quantitative data were integrated through joint displays, bridging results using visual displays to connect major themes [51]. The combined findings provided an in-depth and granular understanding of key factors that were necessary to address in the development and refinement of PrEP4Her, as well as in the development of implementation strategies to support the integration of PrEP services into routine gynecological care. The PrEP4Her pilot was implemented from January 29, 2024, to August 16, 2024. The data gathered during the program are being used toward assessing the program’s efficacy by measuring PrEP4Her’s reach, effectiveness, adoption, acceptability, feasibility, and fidelity. The findings obtained would help guide the implementation of PrEP4Her at a larger scale.

## Discussion

### Anticipated Findings

The quantitative and qualitative data gathered to inform the development of PrEP4Her indicated a need to integrate PrEP services into an already existing and stretched gynecology clinic workflow by optimizing medical assistants and apps to aid in sexual history screening and provision of PrEP services. In addition, there was a gap in knowledge around PrEP as an HIV prevention option for cisgender women and the desire among Black cisgender women to have sexual health and PrEP services integrated into routine gynecology care. This information helped guide the PrEP4Her program implementation in the most effective manner.

PrEP is a highly effective method to decrease HIV acquisition among cisgender women. However, its impact to improve HIV outcomes among women has been hindered due to poor understanding of how to effectively identify strategies to improve uptake among cisgender women. Previous studies conducted at family planning clinics, aiming to improve PrEP uptake, found that although many cisgender women were willing to start PrEP, only a few obtained a prescription for PrEP, highlighting a gap between PrEP knowledge delivery and PrEP uptake. While cisgender women are disproportionately impacted by the HIV epidemic, Black cisgender women living in the Southern United States face heightened challenges such as decreased access to health care services and structural racism, which leads to medical mistrust and greater bias when engaging with providers [52]. Keeping these inequities in mind, the study aims to improve PrEP prescription in this population. We designed a novel intervention, PrEP4Her, which will be focused on increasing PrEP uptake as an effective HIV prevention tool among Black cisgender women by improving access and expanding provider options. Given the pilot site’s Southern US location and the patient population served, preliminary results from this pilot will provide substantial guidance on the successful refinement and implementation of PrEP4Her in similar clinical-care settings across the South. In addition, sampling of gynecological providers across the Southeast United States allowed for greater generalizability of our implementation strategy to other Southern US states that have similar contexts (eg, access to medical resources, access to transportation, proximity to clinics, socioeconomic status, etc.).

PrEP4Her intervention is a novel approach hypothesized to significantly improve PrEP uptake among Black cisgender women. Completion of this study will result in the development of a culturally tailored strategy to improve uptake of PrEP among Black cisgender women. If PrEP4Her shows feasibility, acceptability, and preliminary efficacy in enhancing PrEP uptake for Black cisgender women, a subsequent R01 application will be prepared to conduct a fully powered implementation trial across multiple gynecology care sites across the South. Even in the absence of preliminary efficacy of the pilot, the knowledge gained around determinants and components to include in the PrEP4Her implementation strategy for integrating PrEP services into gynecological settings will provide novel insights to inform future HIV prevention research among this highly impacted group. The findings obtained from this study will be submitted to peer-reviewed journals for publication and presented at local and national conference meetings. Moreover, to disseminate these findings to the nonacademic community, we are currently in discussion with our patient CAB on the most appropriate method to do so. In addition, we are currently working with our institution’s communication team to ensure that the patient community is made aware of PrEP being offered at the gynecology continuity clinic.

Despite our efforts, our study has a few limitations. It is often challenging to engage clinical care teams to conduct interventions due to workflow demands and time constraints. This may impact the overall efficacy of PrEP4Her due to low adoption and lack of fidelity to PrEP4Her components. To overcome this barrier, investigators will work with leadership in identifying “champions” within the clinic setting to encourage PrEP4Her implementation. Inclusion of a physician practicing in the gynecology continuity clinic as part of our investigative team embeds a committed champion within our pilot site. In addition, recruitment of Black cisgender women will be carried out from our clinical site, to take part in both interviews and quantitative surveys throughout this proposal and this may pose a challenge. To overcome this, we will use well-established research advertising through the university and will also leverage provider referral and referral from our research staff to meet recruitment goals. There is a potential for selection bias, as Black cisgender women who are interested in participating in the study may experience less HIV and PrEP stigma than the general population. To overcome this, efforts will be placed to recruit participants via pamphlets at our clinical site with nonstigmatizing, health-promoting messaging to engage a representative sample. Finally, due to time and budgetary constraints, quantitative evaluation of PrEP adherence with drug level testing via blood or hair for those prescribed, will not be conducted. While this is not currently a primary end point for this study, we will measure PrEP adherence more objectively in a subsequent efficacy trial.

### Conclusion

PrEP4Her intervention, by integrating PrEP services into existing gynecology clinic workflow, is aimed at improving PrEP uptake among Black cisgender women, a population disproportionately impacted by the HIV epidemic. Completion of the intervention will be followed by a multisite type III implementation trial for PrEP service delivery at gynecology clinics across the Southern United States.

## Abbreviations

CAB: Community Advisory Board
PrEP: pre-exposure prophylaxis
RE-AIM: Reach, Effectiveness, Adoption, Implementation, and Maintenance

## Data Availability

The datasets generated during this study or those that will be generated or analyzed in the future will be available from the corresponding author on reasonable request.
